# Supplementary material for: Cortical propagation tracks functional recovery after stroke
Source: PLoS Comput Biol. 2021 May 17;17(5):e1008963. doi: 10.1371/journal.pcbi.1008963 (PMC8159272; doi:10.1371/journal.pcbi.1008963)
Supplement: S1 Appendix — Experimental design: Mice; Surgical Procedures; Motor Training Protocol on the M-Platform; Wide-Field Fluorescence Microscopy; Schallert cylinder test. Signal processing and data analysis: Preprocessing; Event detection; SPIKE-Order; Categorization of events; Three propagation indicators: Duration, Angle, Smoothness. Statistical Tests. (PDF) [file pcbi.1008963.s008.pdf]

S1 Appendix: Materials and Methods

Experimental design

Mice

All experimental procedures were performed in accordance with directive 2010/63/EU on the protection of animals used for scientific purposes and approved by the Italian Minister of Health, authorization n.183/2016-PR. Mice were housed in clear plastic cages under a 12 h light/dark cycle and were given ad libitum access to water and food. We used a transgenic mouse line (C57BL/6J-Tg(Thy1GCaMP6f)GP5.17Dkim/J, referred to as GCaMP6f mice) expressing a genetically-encoded fluorescent calcium indicator under the control of the Thy-1 promoter. Mice were identified by earmarks and numbered accordingly. Animals were randomly assigned to five experimental groups: control, untreated, robot, toxin and combined treatment. Untreated, toxin and combined treatment data were recorded for our previous work ([1]), while control, robot and additional untreated data were derived from a different set of experiments ([2]). Each group contained comparable numbers of male and female mice (weighing approximately 25g). The age of mice was consistent between the groups (ranging from 3 to 4 months).

Surgical Procedures

All surgical procedures were performed under Isoflurane anesthesia (3% induction, 1.5% maintenence, in 1.5L/min oxygen). The animals were placed into a stereotaxic apparatus (Stoelting, Wheat Lane, Wood Dale, IL 60191) and, after removing the skin over the skull and the periosteum, the primary motor cortex (M1) was identified (stereotaxic coordinates 1,75 lateral, 0.5 anterior to bregma). Five minutes after intraperitoneal injection of Rose Bengal (0.2 ml, 10 mg/ml solution in Phosphate Buffer Saline (PBS); Sigma Aldrich, St. Louis, Missouri, USA), the targeted region of the cortex (M1) was illuminated through intact skull for 15 minutes with a white light from an LED lamp (CL 6000 LED, Carl Zeiss Microscopy, Oberkochen, Germany) linked to a 20X objective (EC Plan Neofluar NA 0.5, Carl Zeiss Microscopy, Oberkochen, Germany) to induce unilateral stroke in the right hemisphere. Control mice were injected with 0.2 mL of saline and then illuminate as the others. We choose a photothrombotic stroke model as a non invasive technique to induce a targeted ischemic stroke highly reproducible. 30 days after photothrombosis, a subgroup of animals were perfused first with 20-30 mL of 0.01 M PBS (pH 7.6) and then with 150 mL of Paraformaldehyde 4% (PFA, Aldrich, St. Louis, Missouri, USA). The fixed brains were then cut using a vibrating-blade microtome (Leica, Germany) to obtain 100 μm thick coronal sections that were used for immunostaining of NeuN (1:200, Millipore, Germany). Lesion volume located in the primary motor cortex of the right hemisphere was comparable between animals ($1.2 \pm 0.1 mm^3$, average ± SEM). Botulinum Neurotoxin E (BoNT/E) injections in toxin and combined treatment mice were performed during the same surgical session of the photothrombotic lesions. We used a dental drill to create a small craniotomy over M1 of the healthy hemisphere (ML: -1.75; RC:+0.5). Then 500 nL of BoNT/E were delivered in two separate injections. A cover glass and an aluminum headpost were attached to the intact skull using transparent dental cement (Super Bond, C&S). Afterwards, the animals were placed in their cages until full recovery.

Motor Training Protocol on the M-Platform

Before the first imaging session each mouse was allowed to become accustomed to the apparatus. The animals were trained by means of the M-Platform, which is a robotic system that encourages mice to perform a retraction movement of their left forelimb [1,3]. The task consisted of up to 15 cycles of passive extension of the affected forelimb followed by its active retraction triggered by an acoustic cue. A liquid reward (milk) was delivered at the end of each complete pulling to motivate mice during the training session. The time course of one individual training cycle is detailed in Table 1. All groups performed at least one week (5 session) of daily training. Untreated and toxin mice performed one week of daily training starting 26 days after injury. Control, robot and combined treatment performed four weeks (20 sessions) of daily sessions starting 5 days after surgery and/or photothrombosis; in addition, 5 out of 8 robot mice were also recorded for one week before stroke (5 sessions). The M-Platform was designed to allow mice in all conditions (before stroke, right after stroke, and during the weeks under all treatments) to easily perform the motor task from the very first session by applying similar forces. For the same reason, this robotic device is not suitable to evaluate post-stroke functional impairment.

Status	
0	linear actuator positions forelimb at 10 mm from resting position (passive maximum extension)
1	forelimb remains in extended position (0.50s)
2	acoustic tone (1.00V, 0.50s) signals beginning of task
3	mouse is allowed to perform task of pulling handle back to resting position
4	different acoustic tone (3.00V, 1.00s) marks end of task
5	waiting time before reward supply (0.50s)
6	supply of liquid reward for successful execution of task (0.30s)
7	waiting time (2.00s) to allow mouse to drink reward before next task

Table 1. Time course of training cycle. Each line corresponds to a different value of the status variable.

Wide-Field Fluorescence Microscopy

The custom-made wide-field imaging setup [4–6] was equipped with a 505 nm LED (M505L3 Thorlabs, New Jersey, United States) light was deflected by a dichroic filter (DC FF 495-DI02 Semrock, Rochester, New York USA) on the objective (2.5x EC Plan Neofluar, NA 0.085, Carl Zeiss Microscopy, Oberkochen, Germany). Then a 20x objective (LD Plan Neofluar, 20x/0.4 M27, Carl Zeiss Microscopy, Oberkochen, Germany) was used to demagnify the image onto a high-speed complementary metal-oxide semiconductor (CMOS) sensor (OrcaFLASH 4.0, Hamamatsu Photonics, NJ, USA). The fluorescence signal was selected by a band pass filter (525/50 Semrock, Rochester, New York USA) and images (100 x 100 pixels, pixel size 60 μm) were acquired at 25 Hz. Accordingly, signals from every pixel reflect the activity of hundreds if not thousands of neurons.

Schallert cylinder test

For the evaluation of motor performances mice were placed in a Plexiglas cylinder (7,5 cm diameter, 16 cm height) and, after two minutes of acclimatization, recorded for five minutes by a webcam placed below the cylinder. We analyzed mice spontaneous forelimb use through six time points (before stroke and two days after, and once a week during the four week rehabilitative period). Videos were monitored frame by frame and the spontaneous use of both forelimbs was assessed during exploration of the walls, by counting the number of contacts performed by the paws of the animal. For each wall exploration, the last paw that left and the first paw that contacted the wall or the ground were assessed by an experimenter who was blind to the experimental group. In order to quantify forelimb-use asymmetry displayed by the animal, an asymmetry index was computed, according to Lai et al. 2015 [7]:

$$A = \frac{C_{ipsi} - C_{contra}}{C_{ipsi} + C_{contra}} * 100 \qquad (1)$$

where C_{ipsi} and C_{contra} refer, respectively, to the number of contacts performed with the limb ipsilateral and contralateral to the lesioned hemisphere.

Signal processing and data analysis

Preprocessing

Data acquired during each recording session (one mouse, one day, see Fig 1B) was processed offline using custom routines implemented in Python (Python Software Foundation) and Matlab (MathWorks). Each such dataset consisted of up to 15 cycles of active retraction movements on a slide triggered by passively actuated contralesional forelimb extensions. To ensure the consistency of the field of view across sessions and across mice, each frame of the fluorescence data was offline registered by aligning each frame to two reference points (corresponding to bregma and lambda) that were previously marked on the glass window during the surgery procedure. After the image registration across all days, subjects and groups, the more marginal regions were excluded from the analysis and thus the final area analyzed contains at most a very small portion of the lesion. For the 2D fluorescence data, masking the region of interest and spatial downsampling by a factor 3 for both rows and columns resulted in calcium activity matrices of 12 x 21 pixels. Spatial average over all pixels yielded the mean calcium activity. In parallel, the force applied to the slide by the mouse and the discrete status of the slide were recorded. Using samplings with a time step of 40 ms and acquisition times of up to 400 seconds this yielded recordings with at most 10000 data points. The calcium traces were transformed into z-scores, detrended via subtraction of a moving average of order 75 (three seconds) and, in order to yield a better time resolution, upsampled by a factor 20. This approach was also used in [8].

Event detection

Next, within all of these traces we identified the times of the most relevant discrete events. For the status (Fig 2A) we marked the transition from level 3 to level 4 which corresponds to the completion of the forelimb retraction by the active movement of the mouse upon which the animal received its reward (reward pulling event). For the force (Fig 2B), the mean calcium (Fig 2C) and the individual calcium traces of all the pixels the events are the high-amplitude peaks that can easily be recognized. As peak times we used the upwards crossings of a threshold T which in each of these cases was defined in a data-adaptive manner according to $T = mean(x) + t * std(x)$. The free parameter t

was set to 1.5 for the force and 1.7 for all the calcium traces. In the slower calcium traces, in order to avoid a double detection due to noise, we discarded all peaks that succeeded the previous peak by less than a minimum inter-peak interval of 25 data points (one second).

SPIKE-Order

The peaks (from now on called spikes) of all the pixels can be represented best in a rasterplot like the one shown in Fig 2D. The next important step was to identify the global events that correspond to the peaks of the mean calcium trace. To this aim, we used the cSPIKE-implementation [9] of the SPIKE-Order approach recently proposed in [10] (for detailed mathematical definitions of all the underlying quantities please refer to the Appendix "SPIKE-Order method". The original proposal was designed for rather clean data with well-defined global events. These conditions hold for most of our datasets as well, however, we added a few tailor-made denoising steps that addressed the rare instances of increased noisiness that we observed in some of the datasets.

The procedure consisted of six steps: in an initial denoising step, we filtered out all spikes of individual pixels that were not within 1 second of a mean calcium peak and thus were certainly not part of global events. Secondly, we applied the coincidence detection first introduced for the bivariate measure *event synchronization* [11]. This criterion paired spikes in such a way that every spike was matched with at most one spike in each of the other pixels. Here we combined the original adaptive approach with a maximum allowed distance between spikes of 2.5 seconds. Next, we used the symmetric and multivariate measure SPIKE-Synchronization C [12] to quantify for each spike the fraction of other pixels for whom a matching spike could be found. By setting a threshold value $C_{thr} = 0.75$ we only took into account spikes which were coincident with spikes in at least three quarters of the other pixels, all other spikes were filtered out as background noise.

In a fourth step, we applied the SPIKE-Order D [10] which evaluates the temporal order of the spikes by quantifying for each spike the net-fraction of spikes of other pixels this spike is leading (positive value) or following (negative value). Based on the time profile we identified start and end spikes of global events by tracking the jumps from a negative local minimum (last spike of previous event) to a positive local maximum (first spike of current event). In one further denoising step we discarded split events and eliminated outlier spikes by using a maximum distance between consecutive spikes of 0.15 seconds and thereby kept only continuous global events. The final step used in the visualization of the spike trains in Fig 2D involved the Synfire Indicator [10], a scalar measure which quantifies to what degree the spatiotemporal propagation patterns of the global events are consistent with each other. Optimization of this indicator was used to sort the spike trains / pixels from overall leader to overall follower. Here, overall means that we take into account all global events at the same time. The result is that the first spike trains contain mostly leading spikes, whereas the last spike trains consist largely of trailing spikes.

Note that all parameters of preprocessing, event detection and definition of global events were selected prior to and independently from the subsequent categorization of event and calculation of events. At no point was there an optimization of any of these parameters regarding the results of the SPIKE-order algorithm and the calculation of the three propagation indicators.

Categorization of events

Next, we divided the global events into several types using the following three-level categorization scheme (the corresponding branching structure is shown in Section

Methods): First, we separated all the global events that are not associated with a force peak (non-Force, **nF**). For this we demanded that there is no force peak in the interval [1 second before, 0.75 seconds after] the matching calcium peak. The window was slightly asymmetric to account for the fact that typically force peaks were observed a bit earlier than mean calcium peaks. The remaining force events (**F**) were further subdivided into events that occur during the passive extension of the arm by the slide (Passive, **Pass**) and events that occur outside that window (Active, **Act**). In the passive events the mouse applied force to resist the forelimb extension movement of the robot, whereas the active events were the ones where the force was applied during an active retraction movement (when the status variable was set to 3, i.e. between the Go cue and the completion of the task). Finally, among the active events we distinguished between events which were not completed and thus not rewarded (non-Reward Pulling, **nRP**) and events which lead to a completion of the forelimb retraction and therefore were rewarded with milk (Reward Pulling, **RP**). The categorization criterion was the occurrence of a transition from status 3 to status 4 within [0.75 seconds before, 0.75 seconds after] a calcium peak. This window was symmetric, since the observed temporal distribution of status events was symmetric with respect to the mean calcium peaks.

Three propagation indicators: Duration, Angle, Smoothness

For all global events, the event time was defined as the average time of all the spikes within the event and our first propagation indicator, the event duration, was defined as time from the first to the last spike of the event. To calculate the other two propagation indicators, angle and smoothness, we first generated the propagation matrix by mapping the color-coded relative order of the spikes onto the pixels of the 2D-recording plane (compare Fig 2F). Next, we applied singular value decomposition (SVD, [13]) which searches for spatial patterns by decomposing the propagation matrix P into three simple transformations: a rotation U, a scaling Σ along the rotated coordinate axes and a second rotation V^T.

The rotations U and V^T are orthonormal matrices and Σ is a diagonal matrix containing in its diagonal the singular values σ_i of P. By backprojecting the sorted singular values one at a time

$$\Sigma_1 = \begin{bmatrix} \sigma_1 & & & \\ & 0 & & \\ & & 0 & \\ & & & \ddots \end{bmatrix} \qquad \Sigma_2 = \begin{bmatrix} 0 & & & \\ & \sigma_2 & & \\ & & 0 & \\ & & & \ddots \end{bmatrix}$$

we could obtain various projections of the original propagation matrix

$$P_1 = U\Sigma_1 V^T \qquad P_2 = U\Sigma_2 V^T.$$

The mean gradients with respect to column (c) and row (r) increments of the first two projections were calculated as

$$\begin{cases} g_1^c = \mathbb{E}(-\frac{\partial P_1}{\partial c}) \\ g_1^r = \mathbb{E}(-\frac{\partial P_1}{\partial r}) \end{cases} \qquad \begin{cases} g_2^c = \mathbb{E}(-\frac{\partial P_2}{\partial c}) \\ g_2^r = \mathbb{E}(-\frac{\partial P_2}{\partial r}) \end{cases}$$

with $\mathbb{E}$ denoting the average across pixels while the sign (-) is defined by the directionality in the matrix P going from leader (+1) to follower (-1). The main propagation directions, along the column and row directions,

$$\begin{cases} v^c = \sigma_1 g_1^c + \sigma_2 g_2^c \\ v^r = \sigma_1 g_1^r + \sigma_2 g_2^r \end{cases}$$

were calculated from the weighted average of the mean gradients of the first two projections, with the singular values as weights. Our second propagation indicator, the angle

$$\alpha = \arctan\left(\frac{v^c}{v^r}\right)$$

was defined relative to the horizontal axis.

Finally, our third propagation indicator, the smoothness S, quantified how well the second order approximation, the weighted sum of the projections of only the first two singular values, captures the full spatiotemporal pattern obtained by considering all singular values σ_i. Smoothness is defined as the relative weight of the first two approximations

$$S = \frac{\sigma_1^2 + \sigma_2^2}{\sum_i \sigma_i^2}. \quad (2)$$

This measure is often referred to as the cumulative explained variance or sometimes explained variance ratio of the second order.

Note that these three indicators were developed and selected 'a priori' based on the complementary information they provide about the propagation patterns. No other indicators were tested for their discrimination performance.

Statistical Tests

To asses statistical significant differences in smoothness, duration, and the asymmetry index we employed a mixed-effect model (*R* library *lme4* [14]) for each measure with at most four different factors depending on the analysis of interest: treatment, event type, week, and day. In particular we ran a separate analysis when comparing events type in the healthy group (Fig 4), the effect of stroke during the acute phase (Fig 5), the effect of treatments (Fig 6), the pre-stroke group with the longitudinal control group(S2 Fig), the acute vs the untreated stroke (S3 Fig), and the asymmetry index across treatment groups (S4 Fig). In addition, for each mixed-model considered, initially we started from a full factorial model that considered all possible interactions, such model was then reduced to consider only relevant effects by means of a backward selection with consecutive likelihood-ratio tests. This was done with the function *step* of the *lmerTest* library [15] in order to select a parsimonious feasible model that could decrease the *type I error* rate and increase the statistical power [16]. Once the model parameters were selected, we tested the differences between the least-squares means with the *difflsmeans* function of the *lmerTest* library and then corrected the results with the Holm-Bonferroni correction. For every test we assessed both the normality of residuals and the homogeneity of variance assumptions before reporting the results. We used the Kolmogorov-Smirnov test to check the residual distribution and the Breusch-Pagan test to check the homoscedasticity. If normality assumption did not hold, we adopted a Box-Cox transform of the dependent variable [17]. In case the Breusch-Pagan test revealed a departure from homogeneity of variance, we identified the comparisons that had a significant difference in variance and reported the results, again correcting the p-values with the Holm-Bonferroni method.

For the propagation angle, instead, we adopted the Von-Mises distribution to model the circular characteristic of this indicator and tested the differences in circular variance with multiple Bartlett tests (*equal.kappa.test* function in the *circular* library, [18]). We adopted the Holm-Bonferroni correction to account for multiple comparison bias and we assessed the assumption of Von-Mises distribution with the Watson test [19]. We only reported results of those comparisons for which the Von-Mises distribution held.

References

1. Allegra Mascaro AL, Conti E, Lai S, Di Giovanna AP, Spalletti C, Alia C, et al. Combined Rehabilitation Promotes the Recovery of Structural and Functional Features of Healthy Neuronal Networks after Stroke. Cell Rep. 2019;28(13):3474–3485.e6.

2. Conti E, Scaglione A, de Vito G, Calugi F, Pasquini M, Pizzorusso T, et al. Synergic effect of optogenetic stimulation and motor training boosts recovery of motor functionality after stroke supported by segregation of motor representation. bioRxiv. 2020;2020.03.05.:974972.

3. Spalletti C, Lai S, Mainardi M, Panarese A, Ghionzoli A, Alia C, et al. A robotic system for quantitative assessment and poststroke training of forelimb retraction in mice. Neurorehabilitation and neural repair. 2014;28(2):188–196.

4. Crocini C, Ferrantini C, Coppini R, Scardigli M, Yan P, Loew LM, et al. Optogenetics design of mechanistically-based stimulation patterns for cardiac defibrillation. Scientific reports. 2016;6:35628.

5. Turrini L, Fornetto C, Marchetto G, Müllenbroich M, Tiso N, Vettori A, et al. Optical mapping of neuronal activity during seizures in zebrafish. Scientific reports. 2017;7(1):1–12.

6. Conti E, Mascaro A, Letizia A, Pavone FS. Large Scale Double-Path Illumination System with Split Field of View for the All-Optical Study of Inter-and Intra-Hemispheric Functional Connectivity on Mice. Methods and protocols. 2019;2(1):11.

7. Lai S, Panarese A, Spalletti C, Alia C, Ghionzoli A, Caleo M, et al. Quantitative kinematic characterization of reaching impairments in mice after a stroke. Neurorehabilitation and neural repair. 2015;29(4):382–392.

8. Adam I, Cecchini G, Fanelli D, Kreuz T, Livi R, di Volo M, et al. Inferring network structure and local dynamics from neuronal patterns with quenched disorder. Chaos, Solitons and Fractals. 2020;140:110235. doi:https://doi.org/10.1016/j.chaos.2020.110235.

9. Satuvuori E, Mulansky M, Bozanic N, Malvestio I, Zeldenrust F, Lenk K, et al. Measures of spike train synchrony for data with multiple time scales. J Neurosci Methods. 2017;287:25–38.

10. Kreuz T, Satuvuori E, Pofahl M, Mulansky M. Leaders and followers: Quantifying consistency in spatio-temporal propagation patterns. New Journal of Physics. 2017;19:043028.

11. Quian Quiroga R, Kreuz T, Grassberger P. Event Synchronization: A simple and fast method to measure synchronicity and time delay patterns. Phys Rev E. 2002;66:041904.

12. Kreuz T, Mulansky M, Bozanic N. SPIKY: A graphical user interface for monitoring spike train synchrony. J Neurophysiol. 2015;113:3432.

13. Yanai H, Takeuchi K, Takane Y. Projection Matrices, Generalized Inverse Matrices, and Singular Value Decomposition. Springer; 2011.

14. Bates D, Mächler M, Bolker B, Walker S. Fitting Linear Mixed-Effects Models Using lme4. Journal of Statistical Software. 2015;67(1):1–48. doi:10.18637/jss.v067.i01.

15. Kuznetsova A, Brockhoff PB, Christensen RHB. lmerTest Package: Tests in Linear Mixed Effects Models. Journal of Statistical Software. 2017;82(13):1–26. doi:10.18637/jss.v082.i13.

16. Matuschek H, Kliegl R, Vasishth S, Baayen H, Bates D. Balancing Type I error and power in linear mixed models. Journal of Memory and Language. 2017;94:305–315.

17. Gurka MJ, Edwards LJ, Muller KE, Kupper LL. Extending the Box–Cox transformation to the linear mixed model. Journal of the Royal Statistical Society: Series A (Statistics in Society). 2006;169(2):273–288.

18. Agostinelli C, Lund U. R package `circular`: Circular Statistics (version 0.4-93); 2017.

19. Stephens MA. Use of the Kolmogorov–Smirnov, Cramer–Von Mises and related statistics without extensive tables. Journal of the Royal Statistical Society: Series B (Methodological). 1970;32(1):115–122.
