# Supplementary material for: Cortical propagation tracks functional recovery after stroke
Source: PLoS Comput Biol. 2021 May 17;17(5):e1008963. doi: 10.1371/journal.pcbi.1008963 (PMC8159272; doi:10.1371/journal.pcbi.1008963)
Supplement: S2 Appendix — Adaptive Coincidence Detection; SPIKE-Synchronization; SPIKE-Order; Synfire Indicator. (PDF) [file pcbi.1008963.s009.pdf]

S2 Appendix: SPIKE-Order method

The central method of our analysis is SPIKE-Order, first proposed in [1], which we use to identify global events and to track the propagation patterns within these events by sorting the spikes from leader to follower. This method is time-scale independent since it adapts to the time scale of the dynamic under investigation. It is thus able to deal with the fast propagation patterns in neuronal spike trains in the same way as with the much slower calcium patterns analyzed in the present study. Here we present the more detailed mathematical definitions:

Adaptive Coincidence Detection

Analyzing leader-follower relationships in a spike train set requires a criterion that determines which spikes should be compared against each other. Here we use the adaptive coincidence criterion first proposed in [2]. This coincidence detection is scale- and parameter-free since the maximum time lag $\tau_{ij}^{(m,n)}$ up to which two spikes $t_i^{(m)}$ and $t_j^{(n)}$ of spike trains $m, n = 1, ..., N$ (with N denoting the number of spike trains) are considered to be synchronous is adapted to the local firing rates according to

$$\begin{aligned}\tau_{ij}^{(m,n)} = \min\{&t_{i+1}^{(m)} - t_i^{(m)}, t_i^{(m)} - t_{i-1}^{(m)},\\ &t_{j+1}^{(n)} - t_j^{(n)}, t_j^{(n)} - t_{j-1}^{(n)}\}/2.\end{aligned} \tag{1}$$

SPIKE-Synchronization

Following [3], we apply the adaptive coincidence criterion in a multivariate context by defining for each spike i of any spike train n and for each other spike train m a coincidence indicator

$$C_i^{(n,m)} = \begin{cases} 1 & \text{if } \min_j(|t_i^{(n)} - t_j^{(m)}|) < \tau_{ij}^{(n,m)} \\ 0 & \text{otherwise.} \end{cases} \tag{2}$$

which is either one or zero depending on whether this spike is part of a coincidence with a spike of spike train m or not. This results in an unambiguous spike matching since any spike can at most be coincident with one spike (the nearest one) in the other spike train.

Subsequently, for each spike of every spike train a normalized coincidence counter

$$C_i^{(n)} = \frac{1}{N-1} \sum_{m \neq n} C_i^{(n,m)} \tag{3}$$

is obtained by averaging over all $N - 1$ bivariate coincidence indicators involving the spike train n.

In order to obtain a single multivariate SPIKE-Synchronization profile we pool the coincidence counters of all the spikes of every spike train:

$$\{C(t_k)\} = \bigcup_n \{C_{i(k)}^{(n(k))}\}, \tag{4}$$

where we map the spike train indices n and the spike indices i into a global spike index k denoted by the mapping $i(k)$ and $n(k)$.

With M denoting the total number of spikes in the pooled spike train, the average of this profile

$$S_C = \begin{cases} \frac{1}{M}\sum_{k=1}^{M} C(t_k) & \text{if } M > 0 \\ 1 & \text{otherwise} \end{cases} \tag{5}$$

yields SPIKE-Synchronization, the overall fraction of coincidences. It reaches one if and only if each spike in every spike train has one matching spike in all the other spike trains (or if there are no spikes at all), and it attains the value zero if and only if the spike trains do not contain any coincidences.

SPIKE-Order

While SPIKE-Synchronization is invariant to which of the two spikes within a coincidence is leading and which is following, the temporal order of the spikes is taken into account by the two indicators SPIKE-Order and Spike Train Order.

The bivariate anti-symmetric SPIKE-Order indicators

$$\begin{aligned} D_i^{(n,m)} &= C_i^{(n,m)} \cdot \mathrm{sign}(t_{j'}^{(m)} - t_i^{(n)}) \\ D_{j'}^{(m,n)} &= C_{j'}^{(m,n)} \cdot \mathrm{sign}(t_i^{(n)} - t_{j'}^{(m)}) = -D_i^{(n,m)}, \end{aligned} \tag{6}$$

where the index j' is defined from the minimum in Eq. 2 as $j' = \arg\min_j(|t_i^{(1)} - t_j^{(2)}|)$, assign to each spike either a 1 or a -1 depending on whether the respective spike is leading or following a coincident spike in the other spike train.

SPIKE-Order distinguishes leading and following spikes, and is thus used for color-coding the individual spikes on the leader to follower scale. But it can also be employed to sort the *spike trains* based on a pairwise analysis. For this we use the cumulative SPIKE-Order matrix

$$D^{(n,m)} = \sum_i D_i^{(n,m)}. \tag{7}$$

This anti-symmetric matrix sums up the orders of coincidences from the respective pair of spike trains only and quantifies how much spike train n is leading spike train m. Hence if $D^{(n,m)} > 0$ spike train n is leading m, while $D^{(n,m)} < 0$ means m is leading n. If the current spike train order is consistent with the synfire property (i.e., it displays consistent repetitions of the same global propagation pattern), we thus expect that $D^{(n,m)} > 0$ for $n < m$ and $D^{(n,m)} < 0$ for $n > m$. Therefore, we construct the overall SPIKE-Order as

$$D_< = \sum_{n<m} D^{(n,m)}, \tag{8}$$

i.e. the sum over the upper right tridiagonal part of the matrix $D^{(n,m)}$.

Synfire Indicator

After normalizing by the overall number of possible coincidences, we arrive at the definition of the Synfire Indicator:

$$F = \frac{2D_<}{(N-1)M}. \tag{9}$$

This measure quantifies to what degree coinciding spike pairs with correct order prevail over coinciding spike pairs with incorrect order, or in other words, to what extent the spike trains in their current order resemble a synfire pattern. Conversely, the maximization of the Synfire Indicator as a function of the spike train order within a set

of spike trains can be used to sort spike trains from leader to follower such that the set comes as close as possible to a synfire pattern. Denoting the Synfire Indicator for any given spike train index permutation $\varphi(n)$ as F_φ, the optimal (**s**orted) order φ_s is the one resulting in the maximal overall Synfire Indicator $F_s = F_{\varphi_s}$:

$$\varphi_s : F_{\varphi_s} = \max_{\varphi}\{F_\varphi\} = F_s. \tag{10}$$

Whereas the Synfire Indicator F_φ for any spike train order φ is normalized between -1 and 1, the optimized Synfire Indicator F_s can only attain values between 0 and 1. A perfect synfire pattern results in $F_s = 1$, while sufficiently long Poisson spike trains without any synfire structure yield $F_s \approx 0$. For details on the optimization procedure, please refer to [1].

References

1. Kreuz T, Satuvuori E, Pofahl M, Mulansky M. Leaders and followers: Quantifying consistency in spatio-temporal propagation patterns. New Journal of Physics. 2017;19:043028.

2. Quian Quiroga R, Kreuz T, Grassberger P. Event Synchronization: A simple and fast method to measure synchronicity and time delay patterns. Phys Rev E. 2002;66:041904.

3. Kreuz T, Mulansky M, Bozanic N. SPIKY: A graphical user interface for monitoring spike train synchrony. J Neurophysiol. 2015;113:3432.
