# Supplementary material for: Cortical propagation tracks functional recovery after stroke
Source: PLoS Comput Biol. 2021 May 17;17(5):e1008963. doi: 10.1371/journal.pcbi.1008963 (PMC8159272; doi:10.1371/journal.pcbi.1008963)
Supplement: S1 Table — (PDF) [file pcbi.1008963.s010.pdf]

Panel	Indicator	Event type	Group	Diff. type	p-value	
c	Duration	Act-Pass	Control	Variance	0.002	⋆⋆
e	Smoothness	F-nF			0.001	⋆⋆
		RP-nRP			0.001	⋆⋆
g	Angle	F-nF			10^{-10}	⋆⋆⋆
		Act-Pass			10^{-9}	⋆⋆⋆
		RP-nRP			10^{-8}	⋆⋆⋆
