# Supplementary material for: Cortical propagation tracks functional recovery after stroke
Source: PLoS Comput Biol. 2021 May 17;17(5):e1008963. doi: 10.1371/journal.pcbi.1008963 (PMC8159272; doi:10.1371/journal.pcbi.1008963)
Supplement: S2 Table — (PDF) [file pcbi.1008963.s011.pdf]

Panel	Indicator	Event type	Group	Diff. type	p-value	
a	Duration		Control - Acute stroke	Mean	0.003	**
b		F	Control - Acute stroke		0.026	*
		Act-Pass	Control		0.042	*
		Act	Control - Acute stroke		0.034	*
		Pass	Control - Acute stroke		0.035	*
		RP	Control - Acute stroke		0.014	*
		nRP	Control - Acute stroke		0.028	*
c	Smoothness		Control - Acute stroke		0.007	**
d		F - nF	Control		10^{-10}	***
		F	Control - Acute stroke		0.004	**
		Act	Control - Acute stroke		0.01	*
		Pass	Control - Acute stroke		0.002	**
		RP-nRP	Control		0.008	**
		RP	Control - Acute stroke		0.005	**
f	Angle	F - nF	Control	Variance	10^{-7}	$\star\star\star$
			Acute stroke		10^{-5}	$\star\star\star$
		Act - Pass	Control		10^{-4}	$\star\star\star$
			Acute stroke		0.046	$\star$
