# Supplementary material for: Cortical propagation tracks functional recovery after stroke
Source: PLoS Comput Biol. 2021 May 17;17(5):e1008963. doi: 10.1371/journal.pcbi.1008963 (PMC8159272; doi:10.1371/journal.pcbi.1008963)
Supplement: S3 Table — (PDF) [file pcbi.1008963.s012.pdf]

Panel	Indicator	Event type	Group	Diff. type	p-value	
a	Duration		Untreated - Combined	Mean	10^{-4}	***
			Robot - Combined		10^{-5}	***
			Toxin - Combined		0.006	**
b		F	Untreated - Combined		0.001	**
			Robot - Combined		10^{-5}	***
			Toxin - Combined		0.035	*
		nF	Untreated - Combined		10^{-6}	***
			Robot - Combined		10^{-7}	***
			Toxin - Combined		0.002	**
		F-nF	Untreated		0.037	*
			Robot		0.020	*
		Act	Untreated - Combined		0.002	**
			Robot - Combined		10^{-5}	***
		Pass	Untreated - Combined		10^{-4}	***
			Robot - Combined		10^{-4}	***
			Toxin - Combined		0.01	*
		Act-Pass	Toxin		0.015	*
		RP	Untreated - Combined		0.006	**
			Robot - Combined		10^{-5}	***
		nRP	Untreated - Combined		10^{-4}	***
			Robot - Combined		10^{-5}	***
			Toxin - Combined		0.003	**
		RP-nRP	Toxin		0.006	**
c	Smoothness		Untreated - Toxin		0.029	*
			Untreated - Combined		10^{-5}	***
			Robot - Combined		10^{-4}	***
			Toxin - Combined		0.003	**
d		F	Untreated - Combined		10^{-5}	***
			Robot - Combined		10^{-4}	***
			Toxin - Combined		0.019	*
		nF	Untreated - Combined		10^{-5}	***
			Robot - Combined		10^{-4}	***
			Toxin - Combined		10^{-4}	***
		F-nF	Robot	Variance	0.007	⋆⋆
			Toxin	Mean	0.015	*
		Act	Untreated - Combined		10^{-4}	***
			Robot - Combined		10^{-4}	***
			Toxin - Combined		0.023	*
		Pass	Untreated - Combined		10^{-5}	***
			Robot - Combined		10^{-4}	***
			Toxin - Combined		0.013	*
		RP	Untreated - Combined		10^{-5}	***
			Robot - Combined		10^{-5}	***
			Toxin - Combined		0.005	**
		nRP	Untreated - Combined		10^{-5}	***
			Robot - Combined		10^{-4}	***
			Toxin - Combined		0.010	*
e	Angle		Untreated - Robot	Variance	0.018	⋆
			Robot - Toxin		0.005	⋆⋆
f		F	Untreated - Robot		0.037	⋆
			Robot - Toxin		0.003	⋆⋆
		F-nF	Robot		10^{-10}	⋆⋆⋆
		Act	Untreated - Robot		0.001	⋆⋆
			Robot - Toxin		10^{-4}	⋆⋆⋆
		RP	Untreated - Robot		10^{-5}	⋆⋆⋆
			Robot - Toxin		10^{-4}	⋆⋆⋆
