# Supplementary material for: Cortical propagation tracks functional recovery after stroke
Source: PLoS Comput Biol. 2021 May 17;17(5):e1008963. doi: 10.1371/journal.pcbi.1008963 (PMC8159272; doi:10.1371/journal.pcbi.1008963)
Supplement: S4 Table — (PDF) [file pcbi.1008963.s013.pdf]

Panel	Indicator	Event type	Group	Diff. type	p-value	
d	Smoothness	F - nF	Control	Mean	10^{-5}	***
			Pre-stroke		10^{-4}	***
		F	Control - Pre-stroke		0.049	*
f	Angle	F - nF	Control	Variance	10^{-4}	$\star\star\star$
			Pre-stroke		10^{-9}	$\star\star\star$
		Act - Pass	Control		0.012	$\star$
