# Supplementary material for: Cortical propagation tracks functional recovery after stroke
Source: PLoS Comput Biol. 2021 May 17;17(5):e1008963. doi: 10.1371/journal.pcbi.1008963 (PMC8159272; doi:10.1371/journal.pcbi.1008963)
Supplement: S5 Table — (PDF) [file pcbi.1008963.s014.pdf]

Panel	Indicator	Event type	Group	Diff. type	p-value	
c	Smoothness		Acute stroke - Untreated	Mean	0.005	**
d	Smoothness	Act - Pass	Acute stroke		0.038	*
		Act	Acute stroke - Untreated		0.023	*
		Pass			0.016	*
f	Angle	F - nF	Acute stroke	Variance	10^{-5}	$\star\star\star$
			Untreated		0.042	$\star$
