# Supplementary material for: Cortical propagation tracks functional recovery after stroke
Source: PLoS Comput Biol. 2021 May 17;17(5):e1008963. doi: 10.1371/journal.pcbi.1008963 (PMC8159272; doi:10.1371/journal.pcbi.1008963)
Supplement: S6 Table — (PDF) [file pcbi.1008963.s015.pdf]

Indicator	Group	Diff. type	p-value	
Asymmetry index	Untreated - Combined	Mean	10^{-4}	***
	Robot - Combined		10^{-5}	***
	Toxin - Combined		10^{-6}	***
