# Supplementary material for: Cortical propagation tracks functional recovery after stroke
Source: PLoS Comput Biol. 2021 May 17;17(5):e1008963. doi: 10.1371/journal.pcbi.1008963 (PMC8159272; doi:10.1371/journal.pcbi.1008963)
Supplement: S7 Table — (PDF) [file pcbi.1008963.s016.pdf]

Panel	Indicator	Event type	Group	Diff. type	p-value	
a	Duration		Control - Combined	Mean	10^{-4}	***
			Robot - Combined		10^{-6}	***
b		F	Control - Combined		0.002	**
			Robot - Combined		10^{-5}	***
		nF	Control - Combined		0.002	**
			Robot - Combined		10^{-5}	***
		Act	Control - Combined		0.002	**
			Robot - Combined		10^{-4}	***
		Pass	Control - Combined		10^{-4}	***
			Robot - Combined		10^{-5}	***
		RP	Control - Combined		0.003	**
			Robot - Combined		10^{-6}	***
		nRP	Control - Combined		0.002	**
			Robot - Combined		10^{-6}	***
c	Smoothness		Control - Combined		0.025	*
			Robot - Combined		10^{-4}	***
d		F	Robot - Combined		0.001	**
		nF	Control - Combined		0.018	*
			Robot - Combined		0.002	**
		Act	Control - Robot		0.046	*
			Robot - Combined		10^{-6}	***
		Pass	Control - Combined		0.030	*
			Robot - Combined		10^{-6}	***
		RP	Control - Combined		0.029	*
			Robot - Combined		10^{-5}	***
		nRP	Control - Combined		0.014	*
			Robot - Combined		10^{-4}	***
f	Angle	F	Control - Combined	Variance	10^{-8}	$\star\star\star$
			Robot - Combined		10^{-8}	$\star\star\star$
		Act	Control - Combined		10^{-9}	$\star\star\star$
			Robot - Combined		10^{-9}	$\star\star\star$
		Pass	Control - Combined		0.027	$\star$
			Robot - Combined		0.008	$\star\star$
		RP	Control - Combined		10^{-9}	$\star\star\star$
			Robot - Combined		10^{-9}	$\star\star\star$
		nRP	Control - Robot		0.024	$\star$
			Robot - Combined		10^{-5}	$\star\star\star$
