# Supplementary material for: Cortical propagation tracks functional recovery after stroke
Source: PLoS Comput Biol. 2021 May 17;17(5):e1008963. doi: 10.1371/journal.pcbi.1008963 (PMC8159272; doi:10.1371/journal.pcbi.1008963)
Supplement: Attachment — Submitted filename: Plos Computation biology_revision.docx [file pcbi.1008963.s017.docx]

Dear Editor,

please find enclosed the paper “Cortical propagation tracks functional recovery after stroke” by Cecchini et al. that we wish to resubmit for publication in PLOS Computational Biology. We carefully read the reports of the referees and we have modified the paper accordingly. We hope that the manuscript is now suitable for publication.

Best Wishes,

Gloria Cecchini on behalf of all the authors.

-----------------------------------------

**Reviewer #1:** In this paper, the authors applied an original and advanced analysis approach to measure the effect of stroke and post-stroke rehabilitation paradigms on spatiotemporal activation patterns over the cortex in awake mice. Activation patterns were measured with wide-field calcium imaging during forelimb movements in a robotic system. Specific parameters that describe propagation patterns, i.e. duration, smoothness and angle, were shown to be altered after stroke. Cortical activity propagation after stroke was slowed down and more irregular. Combined treatment led to renewed patterns, which were even faster and smoother than before stroke. These results provide new insights into neural recovery processes, although the biological underpinnings remain to be elucidated.

>>>>> We thank the reviewer for this very positive assessment of our work.

Specific comments:

1) The authors claim in the Introduction that functional outcome after stroke is hardly predictable. However, several studies have shown that motor outcome can be well predicted from the degree of motor impairment in the acute stage. This can be assessed with motor functions tests, possibly supplemented with measurement of neurophysiological or neuroimaging biomarkers. These standard methods of monitoring and prediction of motor outcome should be mentioned and discussed in comparison to the authors’ approach of tracking recovery from cortical activity patterns.

>>>>> We agree with the reviewer that the functional outcome after stroke can be predicted by motor function tests together with neurophysiological assays or neuroimaging measures and that our original wording did not take this into account. We removed it from the sentence in the introduction.

Updated text starting at line 5:

“However, the functional outcome, measured as behavioral recovery, depends on multiple factors such as age, lesion size and type, edema formation or inflammation[2,3]”

We also mention that stroke outcome can be predicted from a combination of neurological assays and neuroimaging studies and we explicitly report the difference between our method and other existing methods.

Additional text at line 534

Although the functional outcome after stroke can be predicted on the basis of motor functions tests with or without neurological or neuroimaging assays [2,53], here we test the idea if cortical propagation measures are affected not only by stroke but also by different therapies. In these regards, our study differs from previous ones that have established that the degree of recovery after stroke can be predicted from a baseline assessment of stroke injury, neural function and clinical status [2, 54-56]. Here, instead, we are not making predictions on the recovery based on acute stroke assessment, here we are showing that the time course of the cortical propagation indicators after stroke changes upon different therapies or spontaneous recovery and therefore these measures can be employed as a proxy to assess and monitor recovery. Finally, additional studies are required to check if our propagation measure can be used to predict functional outcome and how they compare to other measures that predict functional outcome in the stroke acute phase.

=========

2) It is not (directly) clear from the Introduction that the study involved experimental stroke studies in mice. Please adjust.

>>>>> Upon the reviewer’s request we now specify in the introduction that the study is done in awake mice.

Updated text starting at line 37:

“In particular, we use our recently proposed SPIKE-order analysis [23] on in-vivo calcium imaging data from the cortex of awake mice to identify global events and to sort the participating regions from first to last (or leader to follower)”

=========

3) Measurements were started in the first week after stroke. This is the subacute post-stroke stage in mice, and not the acute stage. Please correct.

>>>>> Following the reviewer's suggestion, we changed acute to subacute throughout the manuscript.

=========

4) Sample sizes of the experimental groups varied between 3 and 8. Please explain why these were not equal.

>>>>> The reviewer points out that the sample size is different between groups. The explanation of this discrepancy is that the data analysed here were recorded in different sets of experiments from our group, prior to and independently from the design of the current study, where we applied the same animal handling and data collection protocols.

Moreover, in the new version of the manuscript in the supplementary materials subsection ”Mice” we have added the following sentence: “Untreated, toxin and combined treatment data were recorded for our previous work (Allegra Mascaro et al., 2019), while control, robot and additional untreated data were derived from a different set of experiments (Conti E, et al. 2020).”

=========

5) Fig. 1b shows different color-coded symbols. What does the number of the symbols (for example the thirteen brown diamonds for ‘Untr’ at week 4) represent? Please clarify in the figure legend. If animals were measured multiple times, then I recommend to also code for individual animals in the figure.

>>>>> We thank the reviewer for pointing out that this was not clear. Figure 1b shows the recording schedule for each mouse per day. Days of recording move along the horizontal axis, while the vertical axis corresponds to mice divided per group. Therefore the thirteen brown diamonds correspond to a total of thirteen recorded sessions performed by 4 mice (diamonds are aligned along 4 rows) during a week (5 working days).

In the manuscript we have now updated Fig.1 by adding the x- and y- labels. Moreover, we have provided more details in the caption.

=========

6) It is unclear if the lesion area was included in the imaging window. The lesion contains functionally impaired, structurally damaged or lost neurons, which would significantly affect the cortical activity and propagation profiles. Moreover, the extent of the lesion may have differed between time-points and between groups. How did the authors deal with this issue?

>>>>> In the new Figure 1, we show that the position of the focus of irradiation for the photothrombotic stroke, now highlighted by a star (Panel D), is outside the imaging region. We agree with the comment of the reviewer that in the subacute phase the upper part of the field of view included a small region of the cortex affected by the stroke (see also Allegra Mascaro, Conti et al., 2019, Figure 3A). Nevertheless, after the image registration across all days, subjects and groups, the more marginal regions were excluded from the analysis and thus the final area analyzed contains at most a very small portion of the lesion, if any.

We added the following to the Methods section, “Signal processing and data analysis”, “Preprocessing” subsection of the paper. “After the image registration across all days, subjects and groups, the more marginal regions were excluded from the analysis and thus the final area analyzed contains at most a very small portion of the lesion.”

=========

7) Unfortunately, there was no link to the video (S1 Video).

>>>>> We apologise for this inconvenience, we are not sure what happened here. Maybe there was an issue with the online uploading. We will pay particular attention to this during the resubmission.

=========

8) In the Discussion the authors relate their findings to similar results from previous studies. However, discussion of the possible biological underpinnings of disturbed propagation of cortical activity after stroke, and possible mechanisms of recovery in response to rehabilitation treatment, is lacking. The authors should at least speculate on possible biological mechanisms, for example related to changes in neuronal connectivity and transmission, that could explain their findings.

>>>>> Following the reviewer’s suggestion, we added short paragraphs to the discussion on the possible biological mechanism, and in particular on the importance of spatiotemporal patterns of cortical activity both in motor control and in motor recovery:

Section “Cortical propagation features in healthy mice”

“During global events, we observe a large wave traveling across most of the imaged cortex, where a wide population of excitatory neurons is progressively depolarized. Previous studies suggested that wave propagation is relevant to cortical information transfer during movement preparation and execution (Rubino et al., Nat Neurosci 2006, Takahashi et al., Nat Comm 2015). Traveling waves could contribute to cortical processing by determining when and where the cortex is depolarized, in relation to a motor event (Huang et al., Neuron 2010). Also, travelling waves in the motor cortex have been suggested to facilitate sequences of activation in muscle representations in preparation of goal-directed movements (Riehle et al., Front. Neural Circuits 2013). In our study, each of the novel spatiotemporal features of motor-evoked calcium wave propagation we described could encode for specific aspects of the forelimb movement (for example, active vs passive force application).”

Section “Subacute phase after stroke”

“Wave-like propagation (and possibly information transfer) during global events is likely impaired, as indicated by the higher duration of the motor-evoked cortical calcium wave and lower smoothness. Together with our previous findings showing reduced localization of motor-evoked activation in the untreated stroke (Allegra Mascaro et al., Cell Reports 2019), these data suggest a complex dysregulation of information processing and integration across the cortex that possibly give rise to a fragmented motor command.“

Section “Comparison of different rehabilitation paradigms”

“In the chronic phase after stroke, the establishment of a new pattern in the global event could be pivotal in gaining an optimized computation and communication between subsystems of the brain (Ermentrout & Kleinfeld, Neuron 2001). Results show that task-specific spatiotemporal patterns of neuronal activity spreading across the cortex can indeed be modulated, and how this modulation occurs is dependent on the therapeutic approach. [...] The combined rehabilitation may indeed entrain the cortical network on a new spatiotemporal pattern for the global events, where the duration of the travelling wave is shorter and smoother. In a previous study we showed that also rostro-caudal propagation is faster, and this is associated with a higher correlation in the activity of spared motor-associated areas in REHAB mice (Allegra Mascaro et al., Cell Reports 2019). Taken together, these results suggest that a new communication scheme for motor control emerges in REHAB mice, where information transfer is more efficient (small duration) and coherent (increased smoothness), possibly suggesting an improved effective and structural connectivity between distal regions. Future correlative studies could help disentangle the relationship between altered propagation and anatomical connectivity (Allegra Mascaro et al., JBO 2015).“

====================================================================

**Reviewer #2:** This study induced stroke in transgenic mice and used calcium imaging to study cortical activity propagation patterns and their relation to motor function and recovery. It applies a model of spike train dynamical similarity to calcium activity and uses analyses of the outcome of this mode to explain differences in motor performance.

The idea that different patterns of spatiotemporal dynamics could underlie motor recovery and function is important, and the paper asks this question in an interesting way. The data are high quality. And I am especially impressed with the attempt to apply measures of spreading activity that have more complex dynamics than simple time adjacency of activity in different areas (standard “functional connectivity,” FC). The idea of tracking where and how a neural “message” propagates is an intriguing and attractive one. Despite the limits of resolution in calcium imaging, which give us only a gross picture of how a motor “message” travels, I think any new way to trace signals over more than one synapse or population in the awake brain is useful.

>>>>> We thank the reviewer for this very positive assessment of our work.

However, I think the authors should do more to justify the particular dynamical model used here, which centers on signal duration, smoothness, and direction of travel.

First, SPIKE is a somewhat misleading name. I realize there is a history to this usage but it seems inappropriate here. “Events” in this context are misleadingly referred to, following the model’s moniker, as “spikes.” The time resolution in the present study is 1000x lower compared to the recordings for which the method was originally developed (Satuvuori et al., 2017). Maybe call calcium events “pulses” instead (or similar).

>>>>> We thank the reviewer for this important point about the terminology.

SPIKE-Order is indeed just the name of the original method (proposed in Kreuz et al., New J Phys 2017) that we apply here in a slightly adapted variant. The prefix SPIKE is used for two reasons:

- The relatedness of the method to two previously proposed methods SPIKE-distance and SPIKE-Synchronization. One important feature of all these methods is that they are time-scale independent and parameter free. This means that they can be applied to neuronal spike trains but also as easily to neurophysiological data with very different time-scales. In fact, a previous application of SPIKE-synchronization to a calcium-dataset like the ones used in our study can be found in Lassus et al., Scientific Reports 8:17461, 2017.

- Most of these methods were initially applied to spike trains and their focus on spike timing is in contrast to the previously proposed measure ISI-distance (which was sensitive to interspike-intervals). However, SPIKE-order (which shares this focus on spike timing) was immediately illustrated (Kreuz et al., New J Phys 2017) by means of another calcium dataset as well as with an application to climate data of a time scale many orders of magnitude higher (years).

[The article Satuvuori et al. 2017 cited by the reviewer contains an application to neuronal spike trains but deals only with the symmetrical measures ISI-distance, SPIKE-distance, and SPIKE-synchronization, not with SPIKE-order].

In the present article, the SPIKE-order algorithm gets data such as the ones shown in the rasterplot in Fig. 2d as input. It acts as a black-box like module which is ignorant to where the data come from. So once we arrive at the point of the rasterplot it is easiest to consistently use the traditional terminology of ‘spikes’, ‘spike train’ and ‘spike train set’. In our manuscript this use is restricted to the description of the Methods (Fig. 2 and a few paragraphs in the two Appendices).

The problem with any alternative approach is the following:

When we start with the initial calcium traces it would be most straightforward to speak of peak detection and accordingly to consistently use the word “peaks” instead of the word “spikes”. “Spike train” would then become “peak sequences” and a “spike train set” would be a “set of peak sequences”. However, the problems start to occur when we define the global events from the rasterplot. There we often use expressions like “first/last spike of a global event”. Here a phrase such as “first/last peak of a global event” would be misleading since ‘first/last peak’ could easily be read as referring to parts of the envelope of the global event and not as a tiny part of that global event. Similar problems would be encountered with the word “pulse”.

In response to this important point, we checked the manuscript once more and made sure that we use ‘peaks’ in the context of the calcium traces and ‘spikes’ only once we deal with the rasterplot. While the use of ‘spikes’ was already in line with this, we found 19 occurrences in which we now replaced the more general term ‘events’ with ‘peaks’. This avoids any confusion between the ‘events’ in the calcium signals (now consistently called ‘peaks’) and the ‘global events’ that we define from the rasterplot.

In summary, we now consistently use these three distinct terms:

Peaks – the ‘events’ detected in the force traces and in the individual calcium traces as well as in the mean calcium trace (e.g. force peaks or calcium peaks)

Spikes – the small time marks in the rasterplot which are subject to the SPIKE-order analysis

Events – this term alone is now restricted to the discrete status events (which are not peaks) and also used in th the “global events” defined from the rasterplot

=========

However, this is not just a matter of semantics: no evidence is presented suggesting that dynamical spreading serves or obeys the same functional principles at the millisecond scale as at the second scale. I rather suspect they follow different constraints and serve different goals. Certainly, the SPIKE model is a welcome attempt to move past simplistic FC models but I think this needs to be justified more. The model also introduces three new parameters to augment standard FC analysis, along with numerous pre-processing steps. This is the innovation in the paper, but it also a priori makes it more likely that the model will discover differences in the functional data. If the model itself exploited some compelling aspect of the dynamic organization of neural activity (e.g., communication efficiency over the network, etc.), this would be fine. But at present, the model’s application is not well justified. Thus, it’s hard to say whether the model succeeds because it captures something important about dynamical activity leading to successful motor achievement or because it has been tuned to the data via the addition of parameters and/or because of the winnowing of the data in pre-processing.

>>>>> We thank the reviewer for raising this very important point. Our response is threefold:

1. As in our reply to the previous point, we would first like to point out that the SPIKE-order algorithm (Kreuz et al., 2017) is time-scale independent since it adapts to the time scale of the dynamic under investigation. It is thus able to deal with the fast propagation patterns in neuronal spike trains in the same way as with the much slower calcium patterns analyzed in the present study.

However, figuring out whether or not there are different functional principles underlying the dynamics in these two different time scales would require a much more detailed comparative analysis of both of them at the same time. We agree with the reviewer, that it is certainly possible (probably even likely) but either way, we do not think that this affects the validity of the present analysis.

2. The standard FC analysis techniques often rely on some form of pairwise correlation analysis (either symmetric or directional and either in the temporal or in the spectral domain) and the reconstruction of a graph/network, typically based on an adjacency matrix derived from the correlation.

There are a few important differences between these FC techniques and the method used here:

- FC techniques typically average over long time intervals. Here we use a time-resolved approach that is able to characterize individual global events and thus track changes in propagation patterns even over short times.

- FC techniques rely on a pairwise correlation analysis. The initial coincidence detection that we use here is pairwise as well, but the rank ordering of the pixels takes place in the space of all possible pixel permutations which makes the method essentially multivariate instead of bivariate.

- FC techniques typically result in a graph or network describing the relationship between different brain regions. The SPIKE-order algorithm produces two-dimensional matrices which characterize the detailed spatio-temporal propagation during individual global events (see Fig. 2f for an example). These patterns are then characterized by means of three indicators (Duration, Smoothness and Angle). These three indicators were developed and selected a ‘priori’ based on the complementary information they provide about the propagation patterns. No other indicators were tested for their discrimination performance.

Overall, the approach used in this study is certainly very different from the standard functional connectivity techniques. Moreover, it provides results of a very complementary nature. The success of our method with the data analyzed here is very promising, future investigations on other more diverse datasets will show whether this can be generalized.

3. Finally, as already mentioned above, the SPIKE-order algorithm itself is time-scale independent and thus parameter-free. The only parameters that had to be adjusted in our analysis were part of the standard preprocessing (spatial averaging, detrending etc.) and the various event detection schemes (mainly threshold crossing detection in the calcium traces and definition of global events from the resulting rasterplot). The suitability of these choices were then verified by visual inspection of a few randomly selected calcium traces. Only then were the resulting rasterplots given as input into the SPIKE-order algorithm. At no point was there an optimization of any of these parameters regarding the results of the SPIKE-order algorithm and the calculation of the three propagation indicators.

In the manuscript we addressed the first point by adding a sentence in the introduction of the Supplementary Material 2 (“S2 Appendix: SPIKE-Order”):

“This method is time-scale independent since it adapts to the time scale of the dynamic under investigation. It is thus able to deal with the fast propagation patterns in neuronal spike trains in the same way as with the much slower calcium patterns analyzed in the present study.”

Regarding the second point, we extended the first paragraph in the “Comparison with existing methods” Section of the Discussion in which we confront our new approach with the standard FC techniques and describe the aforementioned differences:

“Most analysis tools for wide-field optical images perform simple pairwise correlation and time lag analysis (Pearson correlation and phase synchrony), which are window- and not

event-based as in this study (e.g. Haupt et al., 2017). Functional connectivity techniques typically result in a graph or network describing the relationship between different brain regions (Brier et al., 2019; Vanni et al., 2017). Directionality is explored in Mitra et al., 2018 by using Granger causality, which is dependent on a priori selection of regions of interest (ROIs). Since commonly used analyses tools are based on averaged activity under resting state, important information on single events is missing. This is especially important when considering motor evoked activity, which is directly associated with the execution of single movements (active forelimb pulling in our case). The detailed propagation analysis we showed here would not be possible with the widely used optical flow techniques ...”

Moreover, we added this phrase in the Supplementary Material 1 (“S1 Appendix: Materials and Methods”) at the end of the Subsection “Three propagation indicators: Duration, Angle, Smoothness” within the Section “Signal processing and data analysis”:

“Note that these three indicators were developed and selected a ‘priori’ based on the complementary information they provide about the propagation patterns. No other indicators were tested for their discrimination performance.”

For the third point, in the Supplementary Material 1 (“S1 Appendix: Materials and Methods”) we added a sentence at the end of the Subsection “SPIKE-order” within the Section “Signal processing and data analysis”:

“Note that all parameters of preprocessing, event detection and definition of global events were selected prior to and independently from the subsequent categorization of event and calculation of events. At no point was there an optimization of any of these parameters regarding the results of the SPIKE-order algorithm and the calculation of the three propagation indicators.”

=========

In any case, the ability to distinguish functional outcomes using the present method is hard to discern without attempts at prediction. Differences in the dynamical measures are small and there is a lot of overlap in the distributions for these measures. One could do ridge regressions or similar on individual sessions to see if its group membership can be predicted based on smoothness, duration etc. Without this, I don’t think we can really say these measures constitute a “biomarker.” I would say they are more like average trends or apparent distinctions in dynamics.

>>>>> We agree with the reviewer that, without any regression, the statement on the “biomarker” was a bit of an overstatement. Therefore, we changed the title to “Cortical propagation tracks functional recovery after stroke” and toned down the claims on the spatiotemporal features being biomarkers. Nevertheless, in the Discussion we still mention the possibility of using them as biomarkers in the future:

“In conclusion, our new spatiotemporal propagation indicators could represent promising biomarkers that are able to uncover neural correlates not only of motor deficits caused by stroke but also of functional recovery during rehabilitation. In turn, these insights could pave the way towards more targeted post-stroke therapies.”.

=========

Minor Points:

I’m not sure “smoothness” is the appropriate term for the measure employed here. It seems more like the linearity or maybe the dimensionality of the data. What looks like a dominant pattern of propagation to our eyes—or to an orthogonal transform like SVD—does not necessarily constitute the “smooth” spreading of activity.

>>>>>We agree with the reviewer that smoothness is not a necessary consequence of having an high percentage of variance explained by the first two SVD components. In technical terms what we name smoothness is actually the cumulative percentage of variance explained up to the second order and if this value is high it means that the data can be well represented when using only an approximation up to the second order. We debated the use of an acronym as EVR Explained Variance Ratio as opposed to smoothness which will be better suited technically but in our opinion will make the text harder to follow, especially because in our case it holds that a smoother spread of activity corresponds to a high value of EVR. So we would like to keep using the term smoothness but giving the technical definition in the supplementary material.

Additional text starting at line in the Supplementary Material 1 (“S1 Appendix: Materials and Methods”), line 188:

This measure is often referred to as the cumulative explained variance or sometimes explained variance ratio of the second order.

=========

How big are the calcium signals? Fig 2 gives arbitrary units but these should instead be given as Z-scores.

>>>>> That’s a very good idea. We made the change in Fig. 2 and in addition we also changed the scale in Fig. 1c accordingly.

=========

Forgive me if I have misread Fig 5 but the average data (leftmost column) for the controls looks possibly inconsistent with the other columns. I assume the leftmost column is an average across conditions (F/nF, Act/Pass, RP/nRP). If so, I am not certain that the variance could have gone down quite so much compared to the individual conditions. Is it possible that the vertical scales are not the same? Of course, it’s hard to tell by eye.

>>>>> We thank the reviewer for spotting this tricky issue. The vertical scales are indeed the same. The confusion arises from the fact that Fig. 5a is not the average of the data of Fig. 5b. In Fig. 5a each point is the average of all events per day per mouse, while in Fig. 5b each point is the average of all events of a specific type per day per mouse. For this reason mean and standard deviation might vary a lot.

In the manuscript we have now added an explanation in the captions for Figures 4, 5, and 6.

=========

Line 502: I would not call calcium imaging “non-invasive”.

>>>>> In agreement with the reviewer in the new version of the manuscript we have removed the term “non-invasive” .
